# On the trail of Sisyphus – addiction as an existential neurosis?

**DOI:** 10.3389/fpsyt.2023.1243792

**Published:** 2023-08-24

**Authors:** Human-Friedrich Unterrainer

**Affiliations:** ^1^Faculty of Psychotherapy Science, Sigmund Freud University, Vienna, Austria; ^2^Center for Integrative Addiction Research (CIAR), Grüner Kreis Association, Vienna, Austria; ^3^Institute for Religious Studies, University of Vienna, Vienna, Austria; ^4^University Department of Psychiatry and Psychotherapeutic Medicine, Medical University of Graz, Graz, Austria; ^5^Institute of Psychology, University of Graz, Graz, Austria

**Keywords:** work addiction, existential therapy, mythology, addiction, search for meaning, spirituality

## Abstract

In Greek mythology, Sisyphus, king of the Corinthians, dared to deceive the gods and was condemned to roll a boulder to the top of a mountain for all eternity. Shortly before reaching the summit, however, the boulder rolled back down into the valley, and the arduous task had to begin anew. Many of the contents of this classic myth are reminiscent of the therapeutic approach to addictive disorders. In addiction therapy, too, it is often a long and rocky road that ends with a relapse. The therapeutic effort was not entirely in vain, but one often begins to doubt its usefulness. In terms of Sisyphus and a Bio-Psycho-Social Model (BPSM) of health and disease, addiction can be located at the end of a downward spiral. By extension of the BPSM, can addictive disease be considered an expression of existential neurosis? The results of our own research speak in favor of this and show a clearly reduced experience of sense and meaning, but also diminished feelings of hope and forgiveness in addiction patients. However, positive correlations between various parameters of existential well-being and mental health or more appropriate stress coping are also found for the addiction group. These results are supported by neuronal correlates and are mirrored in the general population. Based on this, the consideration of existential issues in addiction therapy can be discussed. Finally, the use of art therapy and work training are proposed as examples of a meaning based therapeutic intervention in dealing with people with addictive diseases.

## Introduction

According to legend, Sisyphus, the king of the Corinthians, was considered the most cunning of his people. He dared to deceive the gods and as punishment was condemned to roll a stone up a mountain for eternity. Just before he reaches the top, however, the stone rolls back down the mountain and the work must begin anew (1). Much of what Sisyphus has to suffer, however, can also be found in the field of addiction [further on, the “addiction” term will be prefered to the now more common term “substance use disorders”; see Schleim et al. (2) for an enhanced discussion]. The drug has to be consumed over and over again – the purpose of this is mostly short-term need satisfaction or to treat other psychological problems. However, finally this dysfunctional form of medication often contributes to the aggravation of the symptomatics (3).

On the other hand, Sisyphus reminds one of the therapeutic efforts regarding drug addiction. Shortly before climbing the peak or achieving long-term abstinence, there is a relapse into old addictive behavior and the stone rolls back down to the valley. Often one is overwhelmed by the hopelessness of the endeavor and the meaningfulness of therapeutic interventions in addictive diseases is more and more questioned with increasing relapse frequency (4). It must be pointed out that a relapse does not mean a completely new start in therapy, especially since it can also be an important indication of what has been neglected or left out of the therapy so far (5). Nevertheless, the way out of an addictive disease is a rocky road to the top of the mountain for the client as well as for the therapist. Only very few people having suffered from an addictive disease, reach the plateau experience (in the sense of Abraham Maslow) (6) of lifelong abstinence, although it should be noted that at least controlled (drug) use is considered a respectable success in many places (7).

### Substance use disorders from a integrative bio-psycho-social perspective

According to the definition of the American Psychological Association (8), addiction may be understood as a state of psychological or physical dependence (or both) on the use of alcohol or other drugs. The term is often used as an equivalent term for substance dependence or substance use disorders and sometimes applied to behavioral disorders, such as gambling addiction. Based on the bio-psycho-social model of health and disease (9), one may now assume that a certain intoxicant (substance-related or non-substance-related), depending on its availability, its mode of action and its tolerability, will encounter the individual with his or her very specific biological and psychological dispostions in further interaction with various environmental factors (for example, through family or circle of friends). In the case of an unfortunate course of events, a downward spiral is set in motion, in which use, abuse, and finally dependence occur, which manifests then more psychologically and/or physically (10).

Correspondingly, there have always been efforts to describe addiction not only as a psychological disorder *per se*, but also as an expression of a deeper underlying disease. For example, Sigmund Freud (who, notably, has never presented his own theory of addiction) already made the remark that “[The success of a treatment for breaking addiction] will only be an apparent one, so long as the physician contents himself with withdrawing the narcotic substance from his patients, without troubling about the source from which their imperative need for it springs…Whenever normal sexual life can no longer be established, we count with certainty on the patient’s relapse” (11, p. 276). Here, on the one hand, one can recognize Freud’s appraisal that it is insufficient to treat addiction only on the symptom level without taking the causes into account. Furthermore, he emphasizes the important role of attachment and relationship behavior (which, from today’s perspective, can be conclusively claimed as part of the term “sexual life”) in the context of the development and course of an addictive disorder. Accordingly, in addition to the predominant learning theory (were addictive behaviors are seen as a kind of dysfunctional coping strategy) and classical psychoanalytic models, concepts for the treatment of addictive disorders based on attachment theory are also prominently discussed in the literature (12, 13).

Another important way of describing addiction is represented by Orford (14), who attempts to characterize addictive disorders as an excessive form of appetite. On the one hand, this approach points to underlying processes common to all addictions; on the other hand, the notion of an addiction continuum, on which we all find ourselves, can serve to remove some of the aura of abnormality from addiction, which can only affect a marginal group in society. Rather, it is much more realistic that addictive disorders affect all levels of society and that every person carries within him or her a potential for addiction that can come to bear in a toxic way when corresponding (negative) life experiences occur. Above all, addictive behavior serves to regulate our emotions. Accordingly, addiction was described by Kantzian (3) as a form of self-medication, in which a dysfunctional attempt is made to stabilize the fragile structure of the personality.

### Experience of meaning as an extension of the bio-psycho-social model

Since the bio-psycho-social model is conceived as a kind of integrative approach, there is the possibility of further extension, e.g., to include a noogenetic or existential dimension, a field of perception which has been most prominently discussed by Yalom (15) within his approach of existential therapy. Furthermore, the consideration of a “meaning”-dimension in psychotherapy is inseparably connected with the name of Viktor Frankl and his epochal work “Der Wille zum Sinn” [Man’s search for meaning] (16), in which man is described as a rational being, and the resulting possibilities of successfully overcoming the most serious existential crises.

Accordingly, Maddi (17) describes an existential neurosis, which can be discussed as an alternative to regular psychiatric diagnoses. Existential neurosis as fueled by an existential vacuum can be characterized primarily by a persistent state of apathy as well as alienation from the real world, whereby feelings of anhedonia (listlessness), anxiety, emptiness, worthlessness, boredom, low self-esteem, and a clouded mood might occur. Subsequently, an attempt can be made to fill this existential vacuum through various (dysfunctional) defense mechanisms, whereby among others an “addictive type” can be described on the one hand, which tends to substance abuse, pathological gambling, excessive eating or pathological buying, or on the other hand the “distraction type,” which imposes an overly stressful daily or work structure on itself or tends to excessive use of entertainment media (18).

Furthermore, there is some empirical evidence for this model, as for instance, Harlow et al. (19) point out that a stressful situation leads to drug use especially when the stressor in question is experienced as meaningless or at least cannot be evaluated as meaningful in hindsight. Conversely, highly stressful situations that are experienced as meaningful can be beneficial to well-being (20). In line with these findings, Antonovsky (21) has presented the model of “Salutogenesis” for the emergence of health as a counter design to the emergence of disease (“Pathogenesis”). This focuses on the question of “how man becomes a good swimmer” (in the sea of life). A central component of the Salutogenesis model is the sense of coherence, which indicates the extent to which life and its processes are experienced as comprehensible, manageable and meaningful. If life is perceived as less coherent, this also manifests itself in deficits in coping with stress.

In line with Frankl (16) as well as with Antonovsky (21), who also described religion as a possible general resistance resource of nourishing the sense of coherence, an immanent (or bio-psycho-social) can be differentiated from a transcendental (spiritual/noogenic) space of perception. Accordingly, the human being makes contact from his bio-psycho-social life-world to a religious/spiritual space of perception. Accordingly, Frankl’s logotherapy and existential analysis primarily take place in the immanent space of perception or the experience of meaning; however, one should “keep the doors to transcendence open” (16, p. 227). Religiosity/spirituality and the relationship to God in Frankl’s sense is described as the experience of an “Über-Sinn” [“Super-meaning”].

### The role of religious/spiritual well-being in addictive disorders

In a next step, I would like to take up the thoughts of Viktor Frankl and link them to the results of our own research. Based on the theoretical concept of a bio-psycho-socio-spiritual realm of perception of the human being, the Multidimensional Inventory of Religious/Spiritual Well-being (MI-RSWB) was developed (22). By means of this measure, six dimensions of subjective well-being are assessed, three of which may apply to the immanent space of perception or so-called “Existential Well-Being” (EWB): Hope Immanent (HI), Forgiveness (FO), Experiences of Meaning and Meaning (SM) and three to the transcendent space of perception or so-called “Religious Well-Being” (RWB): General Religiosity (GR), Connectedness (CO), Hope Transcendent (HT) of perception, respectively (see Ellison (23) for a further theoretical discussion). By summing up all scales, a total “Religious/Spiritual Well-being” (RSWB) score can be formed. Accordingly, RSWB is defined as “…the ability to experience and integrate meaning and purpose in existence through a connectedness with self, others or a power greater than oneself” (24, p. 116). As depicted in Table 1, a description of the content of the subscales can happen most simply on the basis of the respective marker items (with a total of 48 items with 8 items per subscale).

**Table 1:** Dimensions of religious/spiritual well-being from an existential perspective.

Dimension	AoP	Content	Marker item	Opposite
General Religiosity (GR)	T	Religious belief in the traditional sense, which is institutionalized. Affiliation to a religious community	*“My faith gives me a feeling of security.”*	Atheisim, agnosticism
Connectedness (CO)	T	The feeling of being integrated into a larger whole, regardless of a religious community. A spiritual attitude to life	*“I have experienced the feeling of being absorbed into something greater.”*	Isolation
Hope transcendent (HT)	T	The hope for a better life after death or that life after death continues (as opposed to fear of death and dying)	*“I often think about the fact that I will have to leave behind my loved ones.”* (coded reversely)	Existential fear (of death and dying)
Hope immanent (HI)	I	The hope for a more fulfilling life in the future or that things will change for the better	*“I view the future with optimism.”*	Hopelessness
Forgiveness (FO)	I	The ability to forgive yourself or other people or to resign yourself to things that have gone wrong.	*“There are things which I cannot forgive.”* (coded reversely)	Anger, hate
Experiences of sense and meaning (SM)	I	Significant life experiences, for example of honesty and openness, true friendship, loyalty or gratitude	*“I have experienced true (authentic) feelings.”*	Existential vacuum, alienation
Religious/spiritual well-being (RSWB)	I/T	Total score of all six subscales (see also global definition)	–	–

I, Immanent area of perception; T, Transcendent area of perception; AoP, Area of perception; adapted from Unterrainer (25).

The scale has now been successfully validated in several languages so far, and norm values for the Austrian population have also been presented (25). A number of studies have shown that especially the parameters for Existential Well-Being (EWB: HI, FO, BS) are positively related to various parameters of mental health. For example, we observed a moderating positive effect of IWB on the tendency to depressive moods in by tendency insecurely attached adults (up to 40 years in a sample from the normal population) (26). As far as addictive disorders are concerned (27), a generally lower RSWB can be assumed – this applies to all sub-dimensions. However, a positive correlation between the EWB (HI, FO, BS) and sense of coherence [*sensu* Antonovsky; see (21)] as well as active, problem-oriented coping in line with a negative correlation with depressive coping can also be reported for the group of addiction patients. For the dimensions of RWB, these correlations are significantly lower. In another recent study (28), in which data collection took place during the Corona pandemic, this impression was confirmed. The dimensions of EWB [HI and FO; in the abridged version of the MI-RSWB, BS was omitted in favor to get a better model fit; (29)] also proved to be highly significant negative predictors of perceived psychiatric symptom burden as well as substance use in young adults from the normal population. Furthermore, it was possible to show at least a tendency towards neuronal correlates of sensory perception in the brain of addicts. Based on a three-level theory of emotions (primary-secondary-tertiary) represented in Affective Neuroscience (30), the experience of meaning or spiritual connectedness may be neocortically anchored in the area of tertiary or higher emotions. Correspondingly, in a study with poly-drug addicted patients, a negative correlation (*p* < 0.11) between deficits in White Matter structure in the Neocortex and EWB was observed (31).

The Swiss psychoanalyst Jung understood neurosis as suffering of the soul that has not found its meaning. Furthermore he mentioned that one should not look for how to deal with neurosis, but one should find out what it means, what it teaches and what its meaning and purpose is Jung (32). As for the role of taking into account the spiritual dimension or the holy spirit in addiction therapy, Jung (33) in a correspondence with Bill Wilson, one of the co-founders of the group of Alcoholics Anonymous, found a common formula: “Spiritus contra Spiritum,” the holy spirit, against the spirit in alcohol, only one of the two can, according to this methaphor, dwell in the person or one drives out the other.

The success of Anonymous groups in the field of addiction therapy may also lend some empirical evidence to this winged word (34). However, in a clinical study (35), for which the title “Spiritus contra Spiritum” was borrowed, partially contrasting results were found. Here, male alcohol patients in the context of inpatient withdrawal from alcohol were asked about their state of health at two measurement points, at the beginning and end of therapy. There was a significant improvement in the area of EWB. However, there were no significant effects in the area of RWB, caused by this admittedly secular kind of clinical treatment. Restrictively, it must be added that with regard to the decrease in psychiatric symptoms (depression, suicidality), significantly higher effect sizes were shown than with regard to EWB. From these results it can be concluded that a noogenic (meaning-oriented) or religious-spiritual dimension in human perception must also be explicitly addressed in order to experience a change. Although the use of spiritual techniques is widespread, especially in drug treatment, it must also be viewed critically. In fact, drug therapy may be often about accepting that there is no higher power that will lead one from the dark night of the (addicted) soul [*sensu* St. John of the Cross; (36)] back to the shining bright daylight of abstinence – relapse is often pre-programmed with this fantasy. The possibility of slipping into an exaggerated form of religiosity/spirituality in the sense of an addiction shift must also be considered especially for the group of addiction patients. A final evaluation of the role of a religious/spiritual component in addiction therapy is left out at this point [see, e.g., Galanter (37) for further discussion].

## Concluding remarks

### A meaning-based approach in addiction treatment

As already mentioned at the beginning, I understand my remarks as embedded in a bio-psycho-social model of health and disease (in the present case, addictive disorders). In my concluding words I will refer primarily to experiences regarding the possibilities of taking into account a noogenic dimension in the field of inpatient long-term drug treatment and would like to use the Theraepeutic Community (TC) as an example here. The TC has always understood itself to be humanistically based and presents itself as open to all faiths (38). Therefore, the consideration of a noogenic (meaning-oriented) dimension in the treatment program may be discussed despite or because of this. For many people with a former substance use disorder, living together in the community and the possibility of contact associated with it can mean a nourishing experience of existential relevance, which can be seen as an antidote to the loneliness and isolation caused by addiction.

In general, however, I assume that a meaning based treatment approach could be helpful in the field of outpatient drug therapy as well as in more classically oriented psychiatric treatment settings (17, 18). Lastly, I would like to single out two areas here (apart from classic psychotherapy in individual and group settings), which could play a special role in filling the “existential vacuum,” as outlined earlier in this essay. Accordingly, I would like to point to the importance of art as well as occupational therapy within an meaning oriented approach in addiction treatment. The use of creative media such as music, painting, or dance may be effective in promoting positive emotions in the experience of patients diagnosed with a substance disorder (deficits in this area may be considered traditionally characteristic for this patient group). Already in the Greek myth, Orpheus succeeded with the sound of his lyre in persuading Sisyphus to let his stone rest for once and to refrain from his meaningless activity [*cf.* Poltrum (39)]. Finally, I would like to take up the cudgels for occupational therapy (or, less charmingly, “work therapy”) in addiction treatment. So it might make sense sometimes to roll one’s stone up the pyramid of personal needs, freely *sensu* Abraham Maslow (6), in order to reach the longed-for peak experience in the end.

### Sisyphus as a happy man

According to Camus (40), man as a body-soul-spirit being is in a constant state of tension between the inner longing for meaningfulness and the outer meaninglessness of the world. An unsuccessful search for meaning in life can represent a state characterized by feelings of despair and anxiety or chronic aimlessness and apathy. Albert Camus’s philosophical essay “The Myth of Sisyphus” addresses the existential question of whether life is worth living in a universe without meaning or purpose. Despite the eternal punishment of the mythological figure Sisyphus - forever pushing a boulder up a mountain only to have it roll back down - Camus argues that there is a certain beauty and fulfillment in the struggle itself. He contends that even though life may be hopeless and absurd, it is ultimately up to the individual to find his or her own meaning and happiness in that absurdity. Thus, he concludes that Sisyphus can be imagined as happy precisely because of his tireless effort and determination in the face of futility. This concept suggests that the act of striving and the pursuit of personal goals can provide fulfillment and meaning to the human experience, regardless of the ultimate outcome of those efforts – an idea that could also be considered in the treatment of addictive disorders.

## Data availability statement

The original contributions presented in the study are included in the article/supplementary material, further inquiries can be directed to the corresponding author.

## Author contributions

The author confirms being the sole contributor of this work and has approved it for publication.

## Conflict of interest

The author declares that the research was conducted in the absence of any commercial or financial relationships that could be construed as a potential conflict of interest.

## Publisher’s note

All claims expressed in this article are solely those of the authors and do not necessarily represent those of their affiliated organizations, or those of the publisher, the editors and the reviewers. Any product that may be evaluated in this article, or claim that may be made by its manufacturer, is not guaranteed or endorsed by the publisher.
